# Calendar time trends in synchronous metastatic urinary bladder cancer before and after the introduction of immune checkpoint inhibitors: a nation-wide population-based cohort study

**DOI:** 10.3389/fonc.2025.1680916

**Published:** 2025-10-02

**Authors:** Karin Söderkvist, Christel Häggström, Oskar Hagberg, Firas Aljabery, Truls Gårdmark, Lars Holmberg, Abolfazl Hosseini, Tomas Jerlström, Henrik Kjölhede, Per-Uno Malmström, Amir Sherif, Fredrik Liedberg, Anders Ullén

**Affiliations:** ^1^ Department of Diagnostics and Intervention, Oncology, Umeå University, Umeå, Sweden; ^2^ Department of Surgical Sciences, Uppsala University, Uppsala, Sweden; ^3^ Department of Diagnostics and Intervention, Oncology, Register Centre North, Umeå Universtity, Umeå, Sweden; ^4^ Department of Translational Medicine, Lund University, Malmö, Sweden; ^5^ Department of Clinical and Experimental Medicine, Division of Urology, Linköping University, Linköping, Sweden; ^6^ Department of Clinical Sciences, Danderyd Hospital, Karolinska Institute, Stockholm, Sweden; ^7^ School of Cancer and Pharmaceutical Sciences, King’s College London, London, United Kingdom; ^8^ Department of Molecular Medicine and Surgery, Karolinska Institutet, Stockholm, Sweden; ^9^ Department of Urology, School of Medical Sciences, Faculty of Medicine and Health, Örebro University, Örebro, Sweden; ^10^ Department of Urology, Sahlgrenska University Hospital and Institute of Clinical Sciences, Sahlgrenska Academy, University of Gothenburg, Gothenburg, Sweden; ^11^ Department of Diagnostics and Intervention, Urology, Umeå University, Umeå, Sweden; ^12^ Department of Urology, Skåne University Hospital, Malmö, Sweden; ^13^ Department of Oncology-Pathology, Karolinska Institutet, Stockholm, Sweden; ^14^ Department of Pelvic Cancer, Genitourinary Oncology and Urology Unit, Karolinska University Hospital, Stockholm, Sweden

**Keywords:** urinary bladder cancer, population based study, survival trends, metastatic disease, checkpoint inhibitors (ICIs)

## Introduction

For the 3-5% of patients diagnosed with urinary bladder cancer presenting with distant metastases, the five-year survival probability remains below 10% (1–3).

Since the late 1980s, platinum-based combination chemotherapy has been the cornerstone of treatment for metastatic urinary bladder cancer (mUBC) (4, 5). For cisplatin-ineligible patients, carboplatin-gemcitabine was established as an alternative in 2011 (6, 7), and is currently used as first-line chemotherapy in approximately half of the patients treated systemically for mUBC (8). However, platinum-based regimens are associated with a high incidence of serious adverse events (5, 7). Consequently, a substantial proportion of patients with mUBC do not receive any systemic chemotherapy (9). Vinflunine was approved in Europe in 2009 as second-line chemotherapy, though with limited clinical benefit (10, 11).

A new era in the systemic treatment of mUBC was marked by the approval of immune checkpoint inhibitors (ICI) 2017 (12, 13). ICIs demonstrated not only an improved overall survival but also a more favorable toxicity profile. Recent population-based studies have reported improved survival among systemically treated patients with mUBC following the introduction of ICI (14).

Given the historically low uptake of systemic therapy in the real-world setting of mUBC, it remains unclear whether the introduction of immune checkpoint inhibitors (ICI) has translated into improved survival at the population level. As the treatment landscape for mUBC continues to evolve rapidly, benchmarking treatment patterns and survival outcomes in real-world populations is essential to guide clinical practice and policy.

We used the Bladder Cancer Data Base Sweden (BladderBase) 2.0 (15) to investigate survival trends among patients diagnosed with synchronous mUBC between 1997 and 2019 across calendar periods defined by the introduction of novel systemic therapies. We hypothesized that survival in the overall mUBC population improved after the introduction of ICI (2017–2019), due to both the availably of a novel treatment option and an increased proportion of patients eligible for systemic treatment due to ICIs favorable toxicity profile.

## Materials and methods

### Data source and study population

The Swedish National Registry of Urinary Bladder Cancer (SNRUBC) includes comprehensive information on tumor characteristics, treatment and follow-up from virtually all Swedish patients diagnosed with urinary bladder cancer. Patients diagnosed between 1997–2019 in the SNRUBC have been linked to registers at the National Board of Health and Welfare and Statistics Sweden to form BladderBaSe 2.0 (15). The National Patient Register on discharge diagnoses from hospital admissions up to ten years prior to the date of bladder cancer diagnosis was used to calculate the Charlson Comorbidity Index (CCI) based on concomitant diseases, bladder cancer excluded. CCI was categorized into four groups: 0, 1, 2, and ≥3 comorbidities (16, 17). Data on educational level were retrieved from the Longitudinal Integration Database for Health Insurance and Labor Market Studies at Statistics Sweden and categorized into three groups: ≤9 years, 10–12 years, and ≥13 years of education, corresponding to low, intermediate and high education level (18). From Statistics Sweden we also retrieved data on continent of birth and marital status. Date and cause of death were obtained from the Swedish National Cause of Death Register.

This study was approved by The Research Ethics Board at Uppsala University, Sweden (Dnr 2015-277, 2019-03574, 2020-05123, and 2022-01747-02).

For the present study, patients with synchronous distant metastases retrieved from the SNRUBC diagnosis form were selected.

### Variables for analysis

To assess the time trends for overall survival (OS), we categorized calendar years into calendar time periods to reflect general improvements in diagnostic procedures and supportive treatments in general, chemotherapeutic agents available and to facilitate comparisons to the literature. The calendar period 1997–2009 is hereafter denoted as historical. To access differences in survival associated to the introduction of ICI, we further divided the contemporary time period into contemporary pre-ICI (2010–2016) and contemporary post-ICI (2017–2019).

Covariables for adjusted analysis comprise patient factors (age and comorbidity), tumor characteristics (tumor stage based on the TNM Classification of Malignant Tumors as defined by the Union for International Cancer Control, using the editions applicable at the time of diagnosis, grade according to the WHO 1973 grading system from 1997 to 2002 and according to WHO1999 from 2003 and onwards, and histopathology [selected ICD-codes in **Supplementary Table S1**)], as well as and socioeconomic factors (highest education level, continent of birth and marital status).

Systemic treatment was defined on an intention-to-treat basis within six months from date of diagnosis. No additional information on systemic treatment, such as type of systemic treatment (chemotherapy vs. ICI), was registered systematically in SNURBC during the period of the study.

### Statistical methods

Categorical variables are presented with proportions, and continuous variables are presented with median and interquartile range, separated by calendar time periods. All analyses were stratified by sex due to potential differences in treatment patterns and outcomes between men and women (19). Differences in proportions of systemic treatment across calendar periods were tested using Pearson´s Chi-squared test.

In the survival analyses, date of diagnosis of bladder cancer were used as start of follow-up, and date of death, emigration, or 31 dec 2019, as end of follow-up, whatever happened first. Months from diagnosis was used as timescale in the analyses.

Kaplan-Meier analyses were used to estimate survival separated for calendar time periods. As most systemic treatments including ICI are approved in urothelial mUBC only, separate Kaplan-Meier analyses for urothelial mUBC and for non-urothelial mUBC were performed to assess the impact of non-urothelial histopathology on survival estimates. Tests for differences between the calendar time periods were performed with log-rank test and for trend over the calendar time periods with log-rank trend test.

Hazard ratios (HR) based on Cox proportional hazards regression models adjusted for known prognostic patient and tumor factors as well as socioeconomic factors and separated by gender were used to estimate association between calendar year as a continuous variable and time to death. To assess difference in survival between historical and contemporary calendar time periods, Cox proportional hazards regression models adjusted for gender, known prognostic and socioeconomic factors were used. To test our hypothesis, that the introduction of ICI would improve OS, we assessed association between calendar period pre- and post-ICI with the pre-ICI calendar period as reference. Both unadjusted models, and models adjusted for known prognostic factors, and prognostic and socio-economic factors, are presented. The proportional hazards assumption was tested with Schoenfeld residuals, and no violations were found.

All data management and statistical analyses were performed using Stata 17.0 MP (StataCorp LLC., Allen, TX, USA).

## Results

The study population included 1751 patients, of whom 1175 (67%) were men and 576 (33%) women. Mean age at diagnosis was 74 years (SD = 10 years) and 869 (50%) of the population had a CCI score of 0 prior to diagnosis (**Table 1**). Data of socioeconomic and demographic factors are shown in **Supplementary Table S2**.

**Table 1:** Characteristics at diagnosis of patients with mUBC stratified according to calendar time, 1997-2009 (historical), 2010-2016 (contemporary pre-ICI) and 2017-2019 (contemporary post ICI) from in BladderBase2.0.

	1997-2009 Historical	2010-2016 Pre-ICI	2017-2019 Post-ICI
	N=891	N=561	N=299
Gender			
Men	609 (68.4%)	363 (64.7%)	203 (67.9%)
Women	282 (31.6%)	198 (35.3%)	96 (32.1%)
Age, median (IQR)	74.6 (67.6-80.7)	74.8 (67.9-81.0)	75.4 (67.7-80.8)
T stage *			
T≤2	397 (44.6%)	392 (69.9%)	238 (79.6%)
T3-4	436 (48.9%)	137 (24.4%)	44 (14.7%)
TX	58 (6.5%)	32 (5.7%)	17 (5.7%)
N stage **			
N+	303 (34.0%)	213 (38.0%)	106 (35.5%)
N0	175 (19.6%)	189 (33.7%)	143 (47.8%)
NX	413 (46.4%)	159 (28.3%)	50 (16.7%)
Grade			
G1/G2	152 (17.1%)	73 (13.0%)	37 (12.4%)
G3	674 (75.6%)	409 (72.9%)	223 (74.6%)
GX/missing	65 (7.3%)	79 (14.1%)	39 (13.0%)
CCI			
0	478 (53.6%)	256 (45.6%)	135 (45.2%)
1	158 (17.7%)	109 (19.4%)	52 (17.4%)
2	146 (16.4%)	108 (19.3%)	59 (19.7%)
3	109 (12.2%)	88 (15.7%)	53 (17.7%)
Histopathology***			
Urotelial	815 (91.5%)	503 (89.7%)	273 (91.3%)
Other	76 (8.5%)	58 (10.3%)	26 (8.7%)

*10 patients with missing T stage are included in TX

**11 patients with missing N stage are included in NX

***13 patients with missing histopathology are included in other.

IQR, Inter Quartile Range; CCI, Charlson Comorbidity Index.

Data on primary treatment for the entire cohort with mUBC were available for 1711 (98%) of the patients. Of those, 209 men (19%) and 104 women (18%) received systemic treatment. The proportion of patients treated systemically was 18.5% in the historical time period, 16.3% in the contemporary pre-ICI time period, and increased to 25.3% in the post-ICI time period (p=0.0007). Corresponding proportions receiving systemic treatment stratified by gender and calendar time periods are shown in **Supplementary Table S3**.

**Figure 1:**
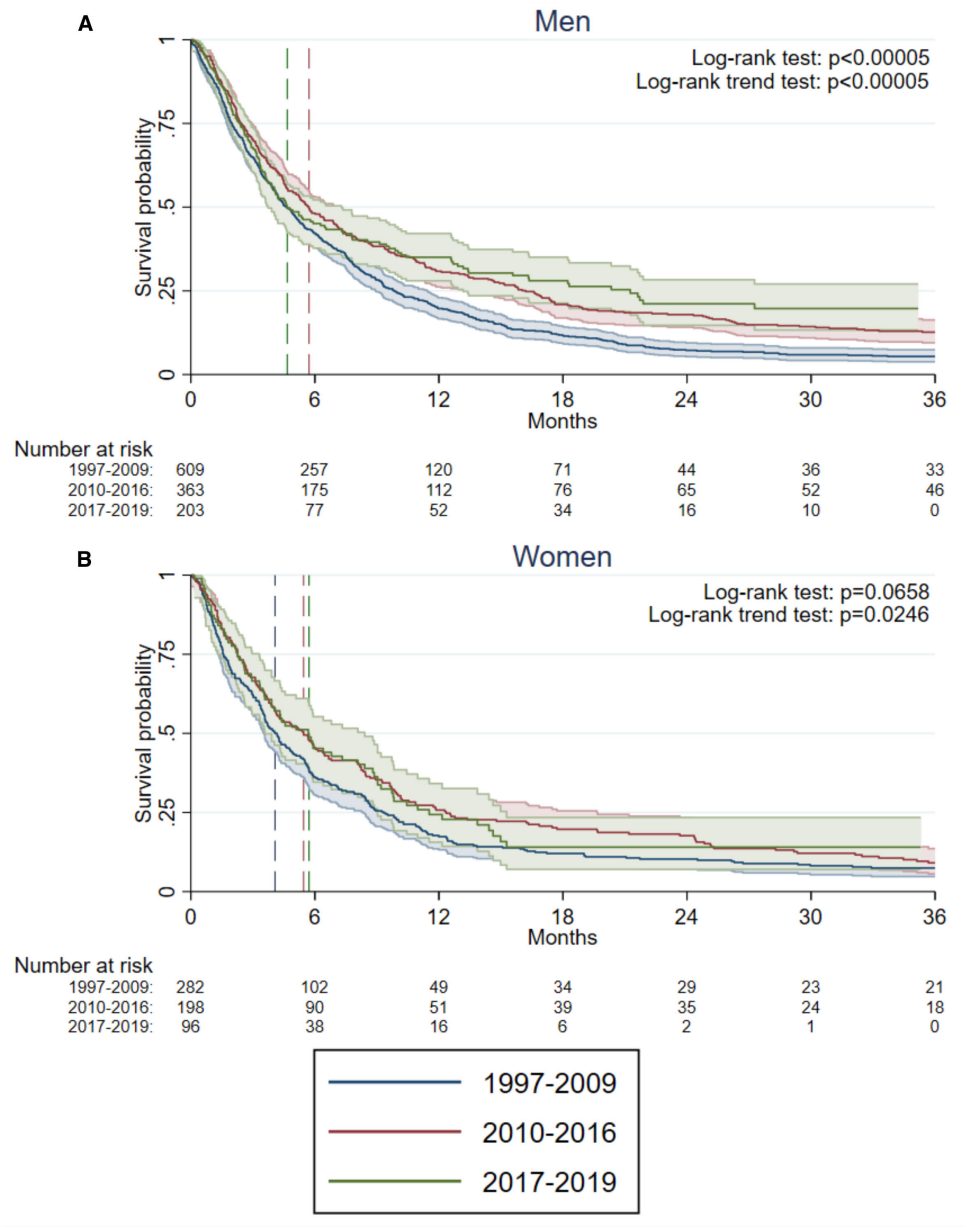
Kaplan–Meier estimates of 3-year overall survival (OS) in **(A)** 1175 men and **(B)** 576 women diagnosed with metastatic urinary bladder cancer (mUBC), stratified by calendar period of diagnosis: historical (1997–2009), contemporary pre-ICI (2010–2016), and contemporary post-ICI (2017–2019), using data from BladderBase2.0. Dashed lines indicate the median OS for each calendar period. In panel **(A)**, the median OS for the historical and post-ICI periods overlap. *P*-values from the log-rank test and log-rank trend test performed to assess differences and trends across calendar periods.

With a median time in follow-up of 4.6 (Inter Quartile Range (IQR) 2.1-11.1) months, 1595 (91%) of the study population had died. The median follow-up time was 4.8 months (IQR 2.1-11.7) for men and 4.3 months (IQR 1.9-10.1) for women. The Kaplan-Meier OS estimates, separated for sex, and for calendar year categories for 36 months of follow-up are visualized in **Figure 1**.

In men, differences in OS were found between calendar time periods (log-rank test p<0.0005). In women, no statistically significant differences between calendar time periods were found (log-rank test p=0.066), but a log-rank trend test showed a statistically significant trend for increased OS over calendar time periods (p=0.025). The OS for patients with urothelial mUBC were comparable to the OS for the entire population (**Supplementary Figure S1a**). The subgroup of mUBC with non-urothelial histopathology comprising only 90 men and 70 women displayed no discernible improvement of OS over time (**Supplementary Figure S1b**).

No statistically significant association to decreased risk of death was found in women using calendar time as a continuous variable (HR 0.99 95% CI 0.97-1.01). In men, each calendar year corresponded to HR = 0.98 (95% CI 0.97-0.99). Using the pre-defined calendar time periods, an association with improved survival was demonstrated for both contemporary calendar time periods compared to the historical time period. In the adjusted analyses a HR = 0.82 (IQR = 0.73-0.93) for the pre-ICI period and a HR = 0.88 (IQR = 0.74-1.04) in the post-ICI period was found for the total cohort with the historical calendar period serving as reference (**Supplementary Table S4**). However, no decreased risk (HR = 1.12 IQR = 0.83-1.51) was demonstrated for the contemporary post-ICI time period compared to the contemporary pre-ICI period (**Table 2**).

**Table 2:** Associations between diagnosis of metastatic urinary bladder cancer (mUBC) during the post-ICI calendar period (2017–2019) and time to overall death, expressed as hazard ratios (HRs) with 95% confidence intervals (CIs), based on data from BladderBase2.0.

	Unadjusted HR (95% CI)	Adjusted Model 1* HR (95% CI)	Adjusted Model 2** HR (95% CI)
Overall cohort***	0.98 (0.83–1.16)	1.05 (0.89–1.24)	1.05 (0.89–1.25)
Men	0.94 (0.77–1.15)	1.01 (0.82–1.23)	1.00 (0.80–1.24)
Women	1.05 (0.80–1.39)	1.13 (0.85–1.51)	1.12 (0.83–1.51)

Analyses are presented for the total cohort and stratified by gender. The pre-ICI calendar period (2010–2016) serves as the reference.

*Model 1 adjusted for age at diagnosis (continuous), grade, N stage, T stage, CCI (in categories) and histopathology

** Model 2 adjusted for all variables in Model 1 and additionally for healthcare region, highest education level, marital status, and continent of birth

***Adjusted for gender

## Discussion

In this nationwide population-based cohort study in patients diagnosed with synchronous metastatic urinary bladder cancer (mUBC) between 1997 and 2019, a survival benefit was observed in the contemporary calendar period compared to the historical period in men and a trend for improved survival in women. Consistent with our hypothesis, we observed an increased proportion of patients receiving systemic therapy in the calendar period following the introduction of immune checkpoint inhibitors (ICI) in 2017. However, looking at the contemporary periods, there was no associated survival benefit in the post-ICI period compared to the pre-ICI period in the adjusted analyses.

There are multiple explanations for the lack of survival benefit associated to the post-ICI calendar period in the present study. The low uptake of systemic treatment in the population is likely a major contributor. Even though an increase in systemic treatment among patients diagnosed with synchronous mUBC was observed, the uptake of systemic treatment was still only one out of four. Despite Sweden being a high-income country with a universal public health insurance, the proportion of patients receiving systemic therapies in this study is in the lower end of what was reported in a recent meta-analysis of systemic treatment in real-world studies of metastatic bladder cancer (9). The authors report a proportion of patients receiving systemic treatment varying between 26-60% in the European setting and 40-85% in studies from the US.

Several factors may contribute to the low proportion of systemically treated patients in our cohort. Given the publicly funded nature of the healthcare system in Sweden, economic barriers are unlikely to be a major contributor. Instead, clinical factors are more plausible explanations. The median age in our cohort was approximately 75 years, and more than one-third of the patients in the contemporary time period (2010–2019) had a Charlson Comorbidity Index (CCI) of 2–3, indicating substantial comorbidity burden. These clinical characteristics likely influence both patient eligibility and physician decision-making regarding systemic therapy. Moreover, patients with metastatic bladder cancer are typically referred to oncology clinics by urologists for evaluation of systemic treatment. This referral process may introduce delays, particularly in the adoption of immunotherapy, as clinical decision-making may still be influenced by prior experiences with the toxicity profile of traditional chemotherapy. Additionally, performance status, patient preferences, and regional variation in oncological practice may further contribute to the observed treatment patterns.

The reported median survival for patients not receiving systemic treatment varied between 2-6.9 months, and the corresponding survival in systemically treated patients were 9,2-34,5 (9). We report survival times in the higher range of non-systemically treated patients. It is reasonable to assume that the low proportion of systemically treated patients in our cohort has strongly influenced the relatively short median survival estimates. Both the proportion of patients receiving systemic treatment and the estimated survival times corresponds well to a recent population based Norwegian study of survival in synchronous metastatic bladder cancer in pre-ICI era (2008–2016) (2), conducted in a country with similar healthcare infrastructure and universal health coverage as Sweden.

One major limitation of this study is the relatively short follow-up period for the post-ICI cohort (2017–2019), which substantially limits the ability to detect long-term survival benefits associated with the introduction of ICI. This is particularly important given the characteristic “tail” in survival estimates associated with ICI, reflecting durable disease control in a subset of responders (22). The short follow-up may therefore underestimate the true impact of ICI on overall survival. Notably, 9% of our population were still alive at the end of follow up, most of which in the post-ICI calendar period, suggesting that longer follow-up could reveal more pronounced survival differences.

Moreover, we cannot rule out that changes in clinical decision-making following the introduction of ICI may have influenced survival outcomes. Response rates for ICI are generally lower than those for platinum combinations (23, 24). One possible minor contributor to the lack of observed survival improvement in the post-ICI period could be a reduced number of patients receiving platinum-based therapy as first-line treatment, due to ICI´s favorable toxicity profile. Factors associated with not receiving systemic treatment include older age, female sex, and impaired performance status (1, 9). The same factors have also been associated with the use of ICI as first-line therapy and a lower likelihood of receiving subsequent treatment lines (1, 2).

The longer median survival observed in men with mUBC as compared to that of women with mUBC is in line with previous population-based reports on non-metastatic muscle-invasive bladder cancer from SNRUBC (20), as well as other published series (19). Patient characteristics were also largely consistent with previous reports (1, 2, 14). As expected, the number of patients with non-urothelial mUBC was low, and no statistically significant improvement in survival over time was observed in this subgroup.

The proportion of females with non-urothelial histopathology align with a SEER-based study on squamous differentiation, which reported this feature to be twice as common in females as in males (21), as well as what has been previously reported from non-metastatic bladder cancer in SNRUBC (20). These histological differences may reflect underlying biological variation between sexes, which could contribute to differences in disease progression and treatment response. In addition to biological factors, gender-based differences in treatment patterns may also contribute to the observed survival gap. Previous studies have shown that women with bladder cancer are less likely than men to receive systemic therapy and to proceed to second-line treatment (9). These disparities may reflect differences in referral practices, clinical decision-making, or perceptions of treatment tolerability, and warrant further investigation.

Another limitation is the absence of detailed data on the specific systemic treatments administered, as well as the number of patients who were initially excluded from systemic treatment but later received it upon disease progression. In Sweden, systemic cancer treatment is administered both on an in-patient and out-patient basis. However, data on these treatments are currently not reliably collected in existing national databases.

A new case report form (CRF) for oncological treatments has recently been introduced within the SNRUBC. An expansion of BladderBaSe 2.0 database is currently underway, incorporating data from 2020 onward. The BladderBaSe 3.0 will therefor include information on specific systemic treatments and subsequent lines of therapy. The absence of detailed treatment data in the present study is unlikely to have affected the observed increase in the proportion of patients receiving systemic therapy during the later calendar period, nor the finding that this increase did not translate into a measurable survival benefit. Given the overall low uptake of systemic treatment and the modest increase in its use following the introduction of ICI, a detectable improvement in survival at the population level may not be reasonable to expect.

## Conclusion

In this nationwide population-based study, we observed an improvement in survival among patients with synchronous metastatic urinary bladder cancer over two decades. However, the introduction of immune checkpoint inhibitors did not result in a measurable survival benefit at the population level. This likely reflects the limited uptake of systemic therapies amongst patients with mUBC. Three out of four patients with synchronous mUBC did not receive systemic treatment at diagnosis, a finding that should be explored in future studies including raising the question whether the same is true for metachronous mUBC.

In summary, no survival benefit was observed following the ICI introduction at the population level, highlighting the need not only for novel therapies but also for improved implementation strategies to ensure equitable access to systemic treatment for all eligible patients.

## Data Availability

The original contributions presented in the study are included in the article/**Supplementary Material**. Further inquiries can be directed to the corresponding author.
