# Supplementary material for: Calendar time trends in synchronous metastatic urinary bladder cancer before and after the introduction of immune checkpoint inhibitors: a nation-wide population-based cohort study
Source: Front Oncol. 2025 Oct 2;15:1680916. doi: 10.3389/fonc.2025.1680916 (PMC12527856; doi:10.3389/fonc.2025.1680916)
Supplement: Supplementary file 1 [file DataSheet1.docx]

**Supplementary figure 1A.** Kaplan–Meier estimates depicting 3-year overall survival (OS) (A) 1,085 men with metastatic urothelial bladder cancer (mUC), and (B) 506 women with mUC, stratified according to year of diagnosis into historical (1997–2009), Pre-ICI (2010–2016) and Post-ICI (2017-2019) within the BladderBase2.0. Corresponding *p*-value from log-rank test in men was <0.00005 and for women 0.1732. The *p*-value for log-rank trend test in women was 0.0719.

**Supplementary figure 1B.** Kaplan–Meier estimates depicting 3-year overall survival (OS) (A) 90 men with other histopathology, and (B) 70 women with other histopathology, stratified according to year of diagnosis into historical (1997–2009), Pre-ICI (2010–2016) and Post-ICI (2017-2019) within the BladderBase2.0. No significant differences were found with the log-rank test.
