# Supplementary figures and images for: Calendar time trends in synchronous metastatic urinary bladder cancer before and after the introduction of immune checkpoint inhibitors: a nation-wide population-based cohort study
Source: Front Oncol. 2025 Oct 2;15:1680916. doi: 10.3389/fonc.2025.1680916 (PMC12527856; doi:10.3389/fonc.2025.1680916)

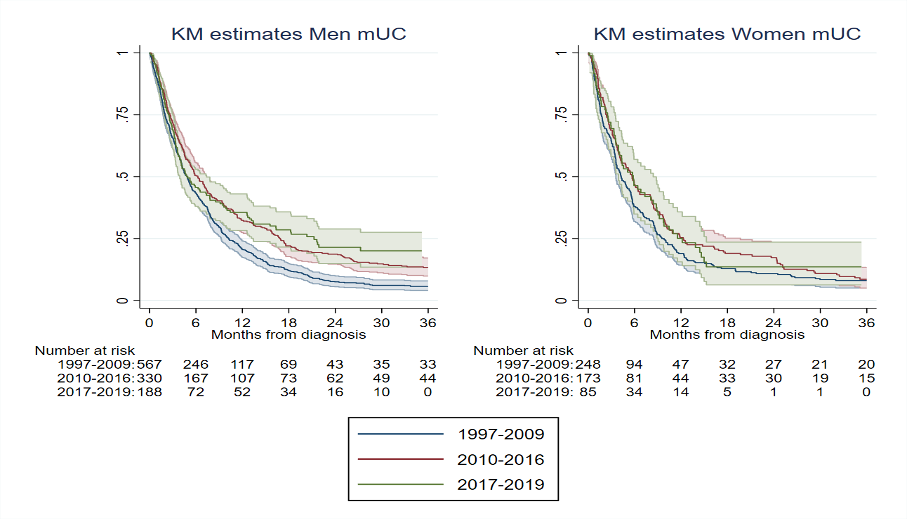

Supplement: Supplementary file 2 [file Image1.png]

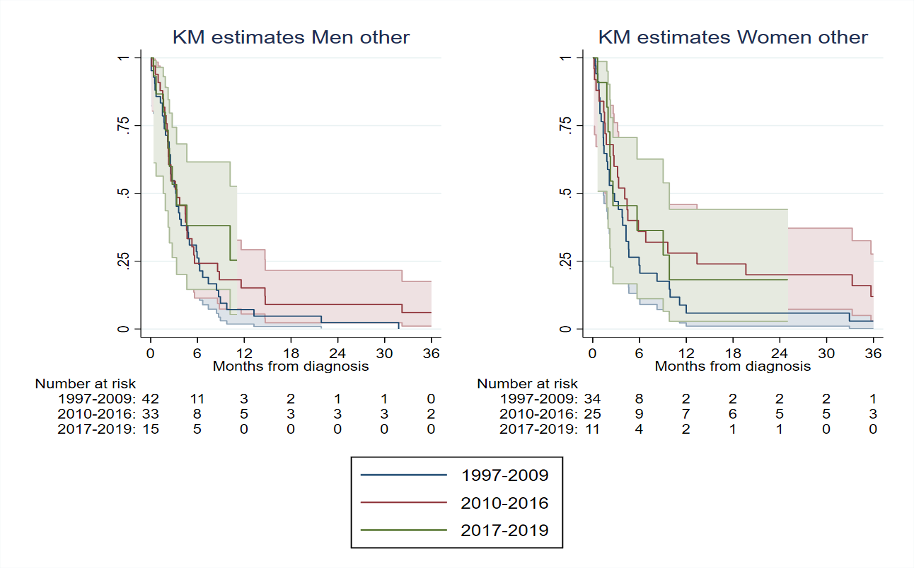

Supplement: Supplementary file 3 [file Image2.png]
