# Supplementary material for: Calendar time trends in synchronous metastatic urinary bladder cancer before and after the introduction of immune checkpoint inhibitors: a nation-wide population-based cohort study
Source: Front Oncol. 2025 Oct 2;15:1680916. doi: 10.3389/fonc.2025.1680916 (PMC12527856; doi:10.3389/fonc.2025.1680916)
Supplement: Supplementary file 4 [file Table1.docx]

**Supplementary Table 1.** SNOMED codes used to categorize the histopathological subtypes

| **Histopathological subtype** | **SNOMED codes** |
| --- | --- |
| Urothelial: | 80203, 81202, 81203, 812031, 812032, 812033, 81223, 81301, 81302, 813021, 813022, 813023, 81303, 813031, 813032, 813033 |
| Other: | |
| Squamous cell carcinoma | 80703, 80103 |
| Adenoid carcinoma | 81403, |
| Neuro-endocrine differentiation | 82403, 82463, 84803, 85603, |
| Unspecified | 80133, 80213, 80413, 80000, 80123, 81201, 87203, 88013, 88303, 88303, 89103, 89803 |
