# Supplementary material for: Calendar time trends in synchronous metastatic urinary bladder cancer before and after the introduction of immune checkpoint inhibitors: a nation-wide population-based cohort study
Source: Front Oncol. 2025 Oct 2;15:1680916. doi: 10.3389/fonc.2025.1680916 (PMC12527856; doi:10.3389/fonc.2025.1680916)
Supplement: Supplementary file 5 [file Table2.docx]

**Supplemental Table 2.** Demographic baseline information of the study population separated for calendar time period of diagnosis.

|  | 1997-2009  Historical | 2010-2016  Pre-ICI | 2017-2019  Post-ICI |
| --- | --- | --- | --- |
|  | N=891 | N=561 | N=299 |
| Highest education level |  |  |  |
| Mandatory school | 496 (55.7%) | 259 (46.2%) | 116 (38.8%) |
| High school | 270 (30.3%) | 209 (37.3%) | 120 (40.1%) |
| University | 98 (11.0%) | 88 (15.7%) | 56 (18.7%) |
| Missing | 27 (3.0%) | 5 (0.9%) | 7 (2.3%) |
| Maritial status |  |  |  |
| Unmarried/single | 104 (11.7%) | 84 (15.0%) | 43 (14.4%) |
| Married/registered partners | 460 (51.6%) | 249 (44.4%) | 147 (49.2%) |
| Divorced/widowed | 325 (36.5%) | 228 (40.6%) | 109 (36.5%) |
| Missing | 2 (0.2%) | 0 (0.0%) | 0 (0.0%) |
| Healthcare region |  |  |  |
| Sthlm/gotland | 110 (12.3%) | 79 (14.1%) | 47 (15.7%) |
| South | 198 (22.2%) | 136 (24.2%) | 69 (23.1%) |
| Southeast | 91 (10.2%) | 70 (12.5%) | 40 (13.4%) |
| Mid | 223 (25.0%) | 132 (23.5%) | 71 (23.7%) |
| West | 146 (16.4%) | 90 (16.0%) | 47 (15.7%) |
| North | 122 (13.7%) | 54 (9.6%) | 25 (8.4%) |
| Missing | 1 (0.1%) | 0 (0.0%) | 0 (0.0%) |
