# Supplementary material for: Calendar time trends in synchronous metastatic urinary bladder cancer before and after the introduction of immune checkpoint inhibitors: a nation-wide population-based cohort study
Source: Front Oncol. 2025 Oct 2;15:1680916. doi: 10.3389/fonc.2025.1680916 (PMC12527856; doi:10.3389/fonc.2025.1680916)
Supplement: Supplementary file 6 [file Table3.docx]

**Supplementary Table 3.** Systemic treatment stratified for gender, separated per calendar time period.

|  | 1997-2009  Historical | 2010-2016  Pre-ICI | 2017-2019  Post-ICI |
| --- | --- | --- | --- |
|  | N=594 | N=361 | N=193 |
| Men | 109 (18.4%) | 62 (17.2%) | 48 (24.9%) |
| Women | 52 (18.8%) | 29 (14.6%) | 23 (26.1%) |
