# Supplementary material for: Calendar time trends in synchronous metastatic urinary bladder cancer before and after the introduction of immune checkpoint inhibitors: a nation-wide population-based cohort study
Source: Front Oncol. 2025 Oct 2;15:1680916. doi: 10.3389/fonc.2025.1680916 (PMC12527856; doi:10.3389/fonc.2025.1680916)
Supplement: Supplementary file 7 [file Table4.docx]

**Supplementary Table 4.** Associations between calendar time period and time to overall death expressed as hazard ratios (HRs) with 95% confidence intervals (CIs) based on data from Bladderbase 2.0.  Analyses are presented for the total cohort and stratified by gender. The historical calendar time period (1997-2009) serves as reference. All models adjusted for gender.

| Group | Time period | Unadjusted  HR (95% CI) | Adjusted*  HR (95% CI) | Adjusted **  HR (95% CI) |
| --- | --- | --- | --- | --- |
| Total cohort | Pre-ICI  (2010-2016) | 0.77 (0.69-0.86) | 0.82 (0.73-0.93) | 0.82 (0.73-0.93) |
|  | Post-ICI  (2017-2019) | 0.75 (0.64-0.87) | 0.87 (0.74-1.03) | 0.88 (0.74-1.04) |
| Men | Pre-ICI  (2010-2016) | 0.75 (0.65-0.85) | 0.78 (0.68-0.91) | 0.79 (0.68-0.92) |
|  | Post-ICI  (2010-2016) | 0.70 (0.58-0.85) | 0.81 (0.66-0.99) | 0.83 (0.67-1.02) |
| Women | Pre-ICI  (2010-2016) | 0.81 (0.67-0.98) | 0.85 (0.72-1.07) | 0.85 (0.69-1.05) |
|  | Post-ICI  (2010-2016) | 0.83 (0.64-1.08) | 0.91 (0.74-1.29) | 0.92 (0.68-1.23) |

*Model adjusted for age at diagnosis (continuous), grade, N stage, T stage, CCI (in categories) and histopathology

**Model adjusted all variables in ** and additionally for healthcare region, highest education level, marital status, and continent of birth
